# Heterogeneous Brønsted Catalysis in the Solvent-Free and Multigram-Scale Synthesis of Polyalcohol Acrylates: The Case Study of Trimethylolpropane Triacrylate

**DOI:** 10.3390/molecules29040918

**Published:** 2024-02-19

**Authors:** Massimo Melchiorre, Maria E. Cucciolito, Roberto Esposito, Simone Silvestro, Francesco Ruffo

**Affiliations:** 1 Dipartimento di Scienze Chimiche, Università di Napoli Federico II, Complesso Universitario di Monte S. Angelo, Via Cintia 21, 80126 Napoli, Italy; massimo.melchiorre@unina.it (M.M.); cuccioli@unina.it (M.E.C.); roberto.esposito@unina.it (R.E.); simonesilvestro01@gmail.com (S.S.); 2 ISusChem Srl, Piazza Carità 32, 80134 Napoli, Italy; 3 Consorzio Interuniversitario di Reattività Chimica e Catalisi (CIRCC), Via Celso Ulpiani 27, 70126 Bari, Italy

**Keywords:** acrylates, trimethylolpropane triacrylate, Amberlyst, Amblerlite, Dowex, heterogeneous catalysis, Brønsted acidic catalysis, esterification

## Abstract

This article presents a thorough investigation into the synthesis of trimethylolpropane triacrylate (TMPTA) via the esterification reaction of trimethylolpropane (TMP) with acrylic acid using Amberlite™ 120 IR (H^+^), Amberlyst^®^ 15, and Dowex™ 50WX8 resins as heterogeneous catalysts. Preliminary comparative tests explored the impact of air flow on water removal during the reaction and different acid-to-alcohol molar ratios (3:1, 6:1, or 9:1 mol:mol). The findings revealed that introducing air significantly enhances TMPTA yield and -OH group conversion, particularly at a 6:1 acid-to-alcohol molar ratio. Based on cost considerations, Amberlite™ 120 IR (H^+^) was selected as the preferred catalyst for further optimization. This included evaluating the effect of catalyst loading (10%, 5.0%, and 2.5% *w*/*w*_tot_) and assessing the impact of a pre-drying process on resin efficiency. The study concluded that optimal conditions did not necessitate drying, requiring 120 °C, a catalyst loading of 10% *w*/*w*_tot_, a 4 h reaction time, an acid:alcohol ratio of 6:1 mol:mol, the presence of MEHQ (0.1% mol/mol_AA_), and air bubbling at 6 ± 1 Nl/h. Catalyst recycling was effectively implemented with a slight reduction in catalytic activity over consecutive runs. Furthermore, the study explored a scaled-up system with a mechanical stirrer, demonstrating the potential for multi-hundred grams scale-up. Considerations for optimizing the air flow stripping system are also highlighted. In summary, this study provides valuable insights into designing and optimizing the esterification process for TMPTA synthesis, laying the foundation for potential industrial applications.

## 1. Introduction

Acrylic acid (AA, CH_2_ = CHCO_2_H) is a bulk chemical widely used in several industrial sectors [1,2,3,4,5]. The carboxylic acid moiety of AA can react with alcohols (ROH) to yield the corresponding acrylates (AAO-R, CH_2_ = CHCO_2_R), preserving the terminal double bond and thus their reactivity in polymerization processes. They are commonly used to achieve homopolymers or copolymers in combination with other reactive monomers such as butadiene, styrene, vinyls, acrylamides, and acrylonitrile [6]. These resultant polymers find extensive use within the chemical industry, contributing to the formulation of plastics, coatings, adhesives, paints, and UV-LED inks [7,8].

**Figure 1:**
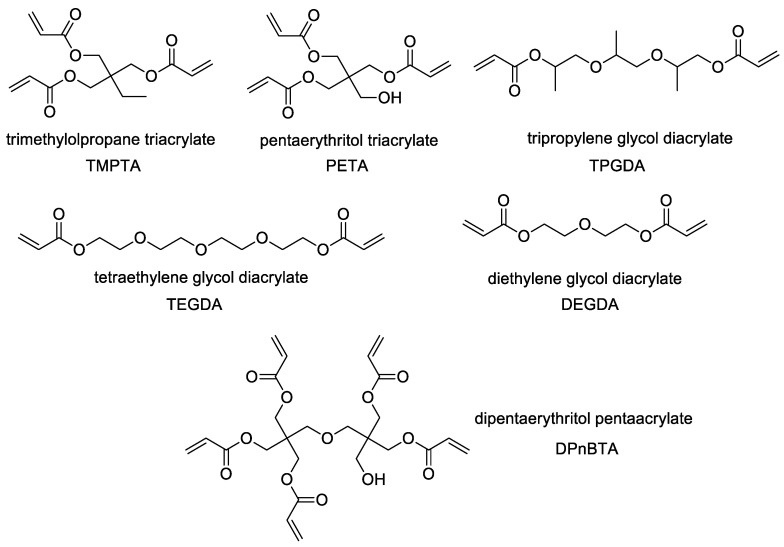
Most common polyalcohol acrylates.

Of particular interest among industrially produced esters are the polyalcohol acrylates, as illustrated in Figure 1.

These acrylic monomers find extensive usage owing to their distinctive properties and reactivity (for use examples: TMPTA [9], PETA [10], TPGDA [11], TEGDA [12], DEGDA [13], and DPnBTA [14]). Several prevalent applications encompass their utility as crosslinking agents in formulating coatings, adhesives, and inks alongside serving as hydrophilic additives and adhesion promoters [15,16]. This category of compounds can be synthesized through the esterification process involving acrylic acid esterification with suitable polyols.

**Scheme 1:**
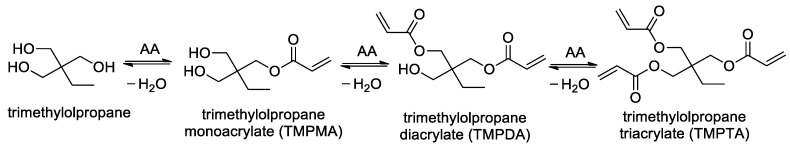
Sequential esterification of acrylic acid (AA) with trimethylolpropane.

Scheme 1 illustrates the synthesis of TMPTA as a representative member within this product family, via intermediate species TMPMA and TMPDA, featuring one and two esterified sites, respectively.

The esterification reaction is an equilibrium, and effective water removal is essential to facilitate sequential ester formation [17]. Elevated temperatures and the presence of a Brønsted acidic catalyst are also necessary to increase the reaction rate. Homogeneous catalysts, including sulfuric acid [18,19], hydrochloric acid [20], or *p*-toluenesulfonic acid [21], have been employed in the esterification of acrylic acid. However, they suffer from several drawbacks, such as necessitating corrosion-resistant equipment, challenges in catalyst–product separation, the use of hazardous solvents (e.g., toluene), propensity for side polymerization reactions, generation of acidic waste, and inherent risks associated with handling highly corrosive substances.

These difficulties can be mitigated through the utilization of heterogeneous catalysts [22,23]. General assessments of homogeneous and heterogeneous Brønsted acid catalysts suggest that homogeneous ones exhibit greater activity, leading to higher acid conversion and product yield under comparable conditions. Nonetheless, although diffusion limitations of reactants within catalyst pores may result in relatively lower conversion for heterogeneous catalysts [24], they offer advantages such as ease of recovery from the reaction system, potential recyclability, and enhanced product purity compared to homogeneous counterparts [25].

Given our interest [26,27,28,29] in devising sustainable catalytic methods for chemical synthesis, this study is centered on the solvent-free esterification of acrylic acid with trimethylolpropane (TMP), utilizing it as the benchmark polyol (Scheme 1). The process employs heterogeneous Brønsted acid catalysts, namely, Amberlite™ 120 IR (H^+^), Amberlyst^®^ 15, and Dowex™ 50WX8. The panel of catalysts was chosen to investigate the effect of different properties like total exchange capacity, particle size, crosslinking degree, and water uptake (parameters reported in Table S1). This investigation accounts for potential upscaling from laboratory to industrial scale, which is a transition that entails challenges in preserving reaction selectivity, efficiency, and safety. Consequently, significant emphasis is placed on avoiding the use of solvents and scrutinizing variables including catalyst loading (and recyclability), reactant molar ratios, the catalyst’s pre-drying conditions, and the effectiveness of water removal. ^1^H NMR investigation was performed to analyze the reaction mixture and evaluate hydroxyl groups conversions and products yields (details in Section 3).

The results herein discussed contribute to the advancement of greener and more efficient chemical synthesis techniques, paving the way for a more sustainable future in the realm of acrylates industrial chemistry.

## 2. Results and Discussion

The selection of temperature is crucial, as it must be maintained within a narrow range: at least 100 °C to facilitate water removal yet not surpassing 120 °C to safeguard the stability of the resin/catalyst. Additionally, given the endothermic nature of acrylic acid esterification [20,30], operating at the highest feasible temperature, namely 120 °C, has been chosen to optimize reaction conversion.

Initially, the ion exchange resin Amberlite™ 120 IR (H^+^) was employed as the acidic heterogeneous catalyst (at 10% *w*/*w*_tot_ relative to total reaction mixture weight). Additionally, 4-methoxyphenol (MEHQ, 0.1% mol/mol relative to acrylic acid) was employed as an inhibitor to impede the spontaneous polymerization of acrylic acid [31] at the given temperature. The hydrophilicity of the reaction mixture tends to trap the water into the liquid phase and favor the instauration of chemical equilibria. Therefore, various approaches involving air flow were evaluated to facilitate the removal of water from the reaction mixture. Air is used, since the presence of molecular oxygen is necessary to properly activate MEHQ, and therefore the use of pure inert gases like nitrogen or argon must be avoided.

Three tests were thus performed in the above conditions with the acid-to-alcohol molar ratio at 3:1 (the stoichiometric ratio): one without air flow, one with surface air flow (venting), and one with sub-surface air flow (bubbling). The results obtained at the 4 h mark (Table 1) are comparable between the first two cases but notably superior in the case involving bubbling.

Because the yield values between the conditions without air flow and venting are similar, the latter technique was not considered for further optimization. Subsequent runs were conducted in a series, varying the acid-to-alcohol molar ratio both with and without bubbling (Table 2). Due to the equilibrium nature of the esterification reaction, the outcomes reveal an increase in TMPTA yield and -OH group conversion with an increasing molar ratio, demonstrating also a more pronounced effect in the presence of bubbling.

While the outcomes from the test involving the 9:1 molar ratio (3 equiv) exhibit slightly higher product yield and -OH groups conversion, the conditions identified as optimal involve the 6:1 molar ratio (2 equiv). These conditions promote a high conversion of -OH groups and yield of TMPTA while employing a lower amount of acrylic acid.

In addition to the Amberlite™ 120 IR (H^+^) resin, the investigation was extended to two other acidic ion exchange resins: Amberlyst^®^ 15 and Dowex™ 50WX8. Despite both resins having a polymeric backbone similar to that of Amberlite™ 120 IR (H^+^) (polystyrene–divinylbenzene), they possess some different properties like total exchange capacity, particle size, crosslinking degree, and water retention. The relevant properties of the three used resins are collected in the Supplementary Materials (Table S1). To facilitate comparison, all three resins underwent pre-drying in an oven to eliminate any water content before utilization. The outcomes of the three reactions performed at 120 °C, employing bubbling, an acid-to-alcohol molar ratio of 6:1, and a catalyst loading of 10% *w*/*w*_tot_, are reported in Table 3.

The results indicate that Amberlyst^®^ 15 is superior to the other two catalysts especially in the initial phase of the reaction. At four hours, only Amberlyst^®^ 15 and Amberlite™ 120 IR (H^+^) promoted almost complete conversion of the OH groups, which stopped at 90% with Dowex™ 50WX8. The different results between the three resins are reasonable due to the different concentration of acidic sites (Dowex 1.1 meq/mL; Amberlite™ 1.8 meq/mL; Amberlyst^®^ 1.7 meq/mL), although these data also indicate that the Dowex™ 50WX8 resin is the most mol-specific active given its lower loading of acidic groups. The macroscopic physical differences between the macroreticular (Amberlyst^®^) and gel resins (Amberlite™) with similar acidic sites concentrations do not seem to play a crucial role.

**Figure 2:**
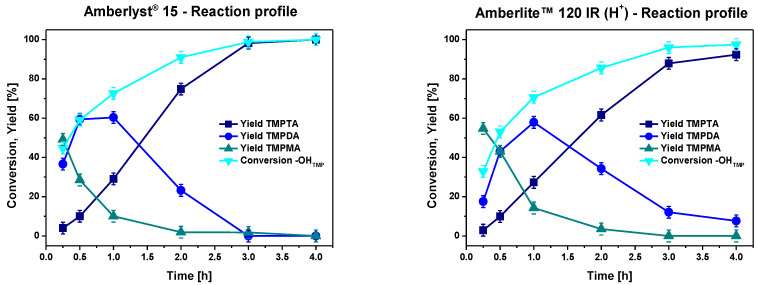
Reaction profile with Amberlyst^®^ 15 (**left**) and with Amberlite™ 120 IR (H^+^) (**right**) at 120 °C, using a bubbling system, acid-to-alcohol molar ratio of 6:1, and a catalyst loading of 10% *w*/*w*_tot_.

To effectively compare the performance of Amberlyst^®^ 15 and Amberlite™ 120 IR (H^+^), their catalytic activity was assessed at various intermediate time points. Full ^1^H NMR spectra and their relevant portions obtained at different times with Amberlite™ 120 IR (H^+^) are reported in Figures S1 and S2. Figure 2 portrays the kinetic profiles of the esterification process, revealing a slightly higher catalytic activity for Amberlyst^®^ 15 compared to Amberlite™ 120 IR (H^+^). Nevertheless, the observed disparity did not present a significant rationale to favor the adoption of Amberlyst^®^ 15 for further optimization endeavors. This is mainly attributed to the substantially elevated cost associated with Amberlyst^®^ 15, which is approximately threefold higher than that of Amberlite™ 120 (H^+^) and therefore less suitable for industrial applications. Consequently, the experimentation was conducted utilizing Amberlite™ 120 (H^+^) as the preferred catalyst of choice for the subsequent phases of the study.

A series of experiments was undertaken to evaluate the efficiency of a resin-drying procedure that causes a mass loss of about 50–55%_wt_ as expected from technical specifications (Table S1, water retention capacity). Although both dry and wet catalysts come to equilibrium with water in the reaction mixture, the possibility of avoiding the pre-drying stage has been examined, as it simplifies the manufacturing of the process. The reaction set was performed, encompassing molar ratios spanning from 3:1 to 9:1. The outcomes obtained at the 4 h mark are detailed in Table 4. To facilitate results comparison, the outputs from reactions set without the pre-drying procedure from Table 2 are also reported herein.

The results suggest that the resin drying process does not confer substantial advantages. From the perspective of industrial scale-up, this presents a favorable outcome as it obviates a potentially energy-intensive step.

Subsequent investigations focused on the impact of catalyst loading. Precisely, a series of three experiments was evaluated at 120 °C utilizing bubbling, an acid-to-alcohol molar ratio of 6:1, and Amberlite™ 120 IR (H^+^) at the following weight percentages: 10% *w*/*w*_tot_, 5% *w*/*w*_tot_, and 2.5% *w*/*w*_tot_. The outcomes of these experiments (Table 5) unveil a trend wherein catalyst loadings below 10% *w*/*w*_tot_ are associated with progressively diminishing yield and conversion values.

To afford the final product TMPTA after the optimized reaction process, the excess of acrylic acid was distilled off under vacuum from the crude mixture. A comparison between the NMR spectra of the purified product and a commercial TMPTA used as a reference is reported in Figure S3. Furthermore, the distillate obtained in the receiving flask was also analyzed by ^1^H NMR (Figure S4), and the recovered acrylic acid was unaffected by any degradation process (e.g., oligomerization, water addition).

**Figure 3:**
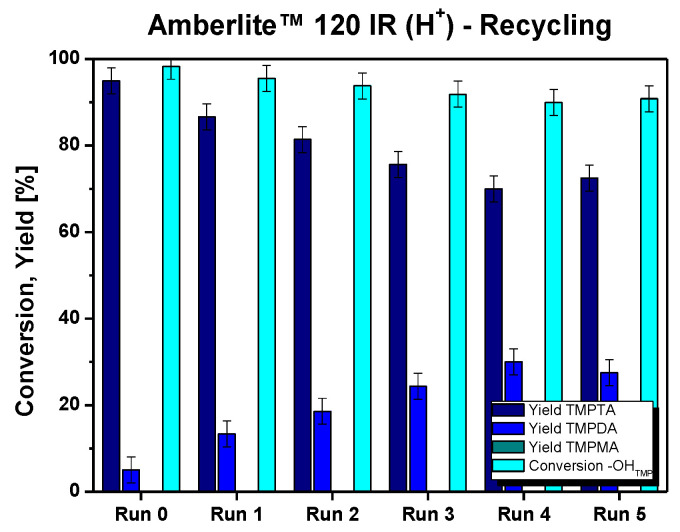
Yields upon sequential catalyst recycles. Conditions: 120 °C, cat. loading 10% *w*/*w*_tot_, 4 h, acid:alcohol 6:1 mol:mol, MEHQ, 0.1% mol/mol_AA_, bubbling system.

Finally, tests were conducted to evaluate the efficacy of the recycled catalyst under the optimized conditions. The results at the 4 h mark, depicted in Figure 3, reveal a consistent conversion trend. However, a slight reduction in catalytic activity is evident, as indicated by the gradual rise in diester formation in comparison to the desired triester product.

A meaningful comparison with existing literature data necessitates a specific focus on systems confronting the challenging esterification of a polyalcohol with acrylic acid. This need arises despite the abundance of both homogeneous and heterogeneous catalysts recognized for their efficacy in esterification [32,33,34]. In alternative studies, 1,3-propanesultone (PS)-functionalized imidazole ionic liquids (ILs) [35] and sulfonated phenylamines [36] are introduced as recyclable acid catalysts, achieving a trimethylolpropane conversion of 100% and TMPTA selectivity of up to 86.4% at 110 °C. The use of a solvent (cyclohexane) and a reducing agent was necessary. The synthesis of TMPTA [37] (and of ditrimethylolpropane acrylate, DTMPA [21]) has been elaborated using *p*-toluenesulfonic acid as a catalyst. Nevertheless, the high temperature (140 °C) and the use of benzene or toluene as solvents raise safety concerns regarding the proposed processes. Other authors have utilized transesterification [38] reactions starting with methyl acrylate. This approach, outside the scope of this study, poses a distinct set of challenges, starting with the necessity to manage the generated methyl alcohol. Dealing with its presence on a large scale demands specific precautions.

Therefore, the advantages of our process lie in avoiding solvents or additives other than the polymerization inhibitor, the ease of catalyst separation, its reusability, and its relatively low cost. However, the optimization of the proposed system required twice the equivalents of acrylic acid and the introduction of stripping apparatus to enhance the yield. Although this is a clear economic disadvantage, in an industrialized scenario, the excess of AA used could be recovered and reused; in fact, the AA–water mixture obtained downstream did not show any degradation process.

### Scale-Up System with a Mechanical Stirrer

**Figure 4:**
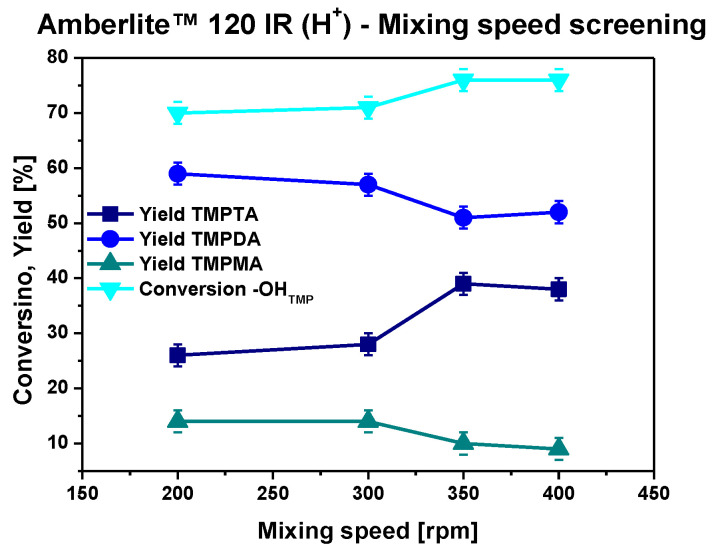
Stirring rate screening with a mechanical stirrer. Reaction conditions: 120 °C, 4 h, cat. 10% *w*/*w*_tot_ acid:alcohol 4.5:1 mol:mol, MEHQ, 0.1% mol/mol_AA_, bubbling system.

To prove the suitability of the developed catalytic system for industrial application, a further scaling-up investigation combined with a mechanical stirrer was carried out. First, a screening of different stirring rates was performed to assess any external mass transfer limitations. Results are collected in Figure 4. Thanks to the comparative nature of this investigation and to avoid any large consumption of reagents, this investigation was performed with a moderate excess of acrylic acid (1.5 equiv), although the most promising performances were achieved using a higher excess (2 equiv) at lower scale.

Stirring at 350–400 rpm is enough to avoid external mass transfer limitations. However, the general trend of yields and conversion was shown to be more comparable with that achieved in stoichiometric conditions (1 equiv of AA) at a lower scale rather than the one obtained in the optimized conditions (2 equiv of AA). Due to this evidence, a new molar ratio screening was performed for the scaled-up system. The relative results collected at 4 h are reported in Table 6.

The general trend was shown to be coherent with the lower-scale investigation; indeed, the increased amount of AA positively affected the TMPTA yield. However, it should be highlighted that the full conversion of TMP hydroxyl groups was not achieved: even with 2 equiv of AA, the conversion reached 83% within 4 h, while at a lower scale, it was almost complete (97%). This evidence is probably due to the reduced efficiency of the used air flow stripping system, which has already been demonstrated to have a high impact on the overall performances of the catalytic system (Table 1 and Table 2). Despite the used flow being almost doubled (from 6 ± 1 to 14 ± 1 Nl/h) in the scaled-up system, the total reactants mass (from 25 to 250 g) and flask volume (from 50 to 500 mL) was ten times higher, probably compromising the overall efficiency. This preliminary evidence demonstrates that multi-hundred grams scale-up is possible provided further specific investigation on the optimization of stripping systems.

## 3. Materials and Methods

Amberlite™ IR 120 (H^+^), Amberlyst^®^ 15, Dowex^™^ 50WX8, anhydrous acrylic acid (AA), trimethylolpropane (TMP), hexamethylbenzene (HMB), and chloroform-d (CDCl_3_) were purchased from Merck KGaA (Darmstadt, Germany) and used without further purification. The trimethylolpropane triacrylate used as a standard product (^1^H and ^13^C NMR in Figures S5 and S6) was kindly provided by Frimpeks TR (Istanbul, Turkey). The heating band was purchased from Watlow Italy Srl (Corsico, Italy) and controlled through a coupled thermocouple and a thermostat. The overhead mechanical stirrer RZR 2051 control was purchased from Heidolph Instruments GmbH & CO. KG (Schwabach, Germany) and equipped with: a PTFE/glass stirring guide and a half-moon impeller, consisting of a single PTFE blade mounted on a calibrated-glass shaft (diameter Ø 10 mm) with PTFE bolt and clamp. The width and height of the clamping area were 6.6 and 2.5 cm, respectively. The stirring position of the blade was fixed at roughly 1 mm from the bottom of the flask. All compounds and reaction mixtures were characterized by NMR spectroscopy with a Bruker Avance Ultrashield 400 (Bruker Corporation, Billerica, MA, USA) or with a Varian 500 Oxford (Varian Inc., Palo Alto, CA, USA), operating, respectively, at a proton frequency of 400 MHz and 500 MHz. NMR samples were prepared using chloroform-d as the solvent and hexamethyl benzene as the internal standard (STD). NMR spectra have been analyzed using MestReNova software (version: 14.3.3).

### 3.1. Catalytic Runs

The reaction setup is illustrated in Figure S7 and comprises the following components: heating/stirring plate, oil bath, reaction flask (50 mL round bottom flask), distillation column equipped with a heating band, thermometer, and distillate receiving flask. The total amount of reagents was set to reach a total volume of 25 mL. The oil bath was fixed at a temperature of 120 °C, whereas the heating band on the column was set to keep the inner temperature at 100 °C. These temperatures were controlled by thermocouples. For the case of venting or stripping set-up, a two-neck flask was used, a purging needle (inner diameter Ø 0.2 mm) was placed in the proper position, and the air flow was regulated at 6 ± 1 Nl/h using a flow meter. Yields and conversions were assessed by suitably integrating the proton NMR spectra of the reaction mixtures as comprehensively described in the experimental section (Section 3.3).

The TMPTA product was purified by distilling an excess of acrylic acid from the hot crude mixture (60 min, 120 °C, 30 mbar) after the reaction was performed in optimized conditions of Entry 1 Table 5 (^1^H NMR spectrum in Figure S3, product distribution TMPTA 92%, TMPDA 7%, TMPMA < 1%).

For the catalyst recycle, each crude reaction mixture was filtered. The resulting catalyst was thoroughly washed with ethyl acetate and dried under vacuum before being used in the successive run. In a typical stoichiometric run, 10.3 g (TMP, 77.1 mmol) of trimethylolpropane, the appropriate amount of acrylic acid (AA, 231 mmol, 16.6 g), catalyst and radical inhibitor (0.1% mol/mol vs. AA, 25 mg) were charged into the flask, which was placed in a preheated oil bath at 120 °C. The total mixture volume was set roughly at 25 mL, and this value was kept constant also in case of different molar ratios to avoid different performance of the stripping apparatus. The reaction was conducted under vigorous magnetic stirring, and the reaction time was considered to be 15 min after the flask was placed into the bath. Sampling from the top of the Claisen adapter was performed to monitor the reaction yield over time.

### 3.2. Scale-Up with Mechanical Stirrer

In the case of the mechanical stirrer, a three-neck round bottom flask was used (500 mL). The three necks were, respectively, equipped with the stripping system (purging needle), the mechanical stirrer, and the distillation apparatus. For a detailed overview, please see Figure S8 in the Supplementary Materials. In a typical experiment, 190 g of AA (1.5 equiv), 79 g of TMP, 26 g of Amberlite™ IR 120 (H^+^), and 0.3 g of MEHQ were consecutively added into the flask, which was then placed into a preheated oil bath (120 °C). The total volume was set to reach 250 mL. The typical stirring rate was set at 400 rpm, the air flow was regulated at 14±1 Nl/h (purging needle inner diameter Ø 1.3 mm), and the heating band was adjusted to keep the inner temperature at 100 °C.

### 3.3. ^1^H NMR Analysis

Approximately 25 mg of each crude mixture is diluted in CDCl_3_ (600 μL) and placed into a cuvette for NMR analysis (500 μL). Before each analysis, 50 μL of a CDCl_3_ solution with HMB 0.1 M was added to each sample as an internal standard (5 μmol).

Hydroxyl groups conversion and products yields (following equations) were evaluated by quantities in mmol obtained from the spectrum (*n_TMP_*, *n_TMPMA_*, *n_TMPDA_*_,_ and *n_TMPTA_*) considering the signal from the standard. Examples of relevant portions of ^1^H NMR over time are reported in Supplementary Materials (Figures S3 and S4).

(1) $$n_{TMP}=\frac{5\;\mu mol}{\int {HMB}_{2.22\;ppm}/18_{H}}\times \int {TMP}_{3.69\;ppm}/6_{H}$$

(2) $$n_{TMPMA}=\frac{5\;\mu mol}{\int {HMB}_{2.22\;ppm}/18_{H}}\times \int {TMPMA}_{4.23\;ppm}/2_{H}$$

(3) $$n_{TMPDA}=\frac{5\;\mu mol}{\int {HMB}_{2.22\;ppm}/18_{H}}\times \int {TMPDA}_{4.14\;ppm}/4_{H}$$

(4) $$n_{TMPTA}=\frac{5\;\mu mol}{\int {HMB}_{2.22\;ppm}/18_{H}}\times \int {TMPTA}_{4.17\;ppm}/6_{H}$$

(5) $${Yield}_{x}\;\%=100\times \left(\frac{n_{x}}{n_{TMP}+n_{TMPMA}+n_{TMPDA}+n_{TMPTA}}\right)$$

with *x* = *TMPMA*, *TMPDA*, *TMPDA* (6)

(7) $${Conversion}_{-OH}\;\%=\frac{1}{3}\times \left[\sum_{i}\left({Yield}_{x}\times i\right)\right]$$

where the integrations symbol (∫) refers to the normalized area of selected peaks in the ^1^H NMR spectra (e.g., “∫ *TMPTA*_4.17_/6*_H_*” refers to the area of the peak at 4.17 ppm attributed to TMPTA, divided by 6, which is the number of protons related to the peak), and *i* is the number of ester groups for each product *x* (e.g., for TMPMA, *i* = 1). An example of ^1^H NMR spectrum analysis is reported in Figure S9.

## 4. Conclusions

In this study, the solvent-free esterification of acrylic acid with trimethylolpropane was comprehensively investigated, focusing on the utilization of commercial heterogeneous Brønsted acid catalysts. The research aimed to address challenges associated with acrylic acid esterification, including the need for efficient water removal, catalyst recovery, and maintaining high selectivity, efficiency, and safety during scale-up from laboratory to industry.

Among the investigated catalysts, Amberlite™ 120 IR (H^+^) emerged as the most suitable, demonstrating competitive activity compared to Amberlyst^®^ 15 while being more economically favorable. The recyclability of the catalyst was evaluated, demonstrating a consistent conversion trend over sequential recycles. Although a slight loss of catalytic activity was observed, the process remained viable, and the catalyst could potentially be reused for multiple cycles.

In conclusion, this work contributes valuable insights into the esterification of acrylic acid with trimethylolpropane, highlighting the significance of catalyst selection, process optimization, and catalyst recycling. The findings pave the way for more efficient and sustainable approaches in acrylic acid ester production, with implications for the broader fields of polymerization, coatings, adhesives, and industrial applications. As the demand for acrylic acid and its derivatives continues to grow, these findings hold promising potential for both economic and environmental advancements.

## Figures and Tables

**Table 1:** Catalytic tests using Amberlite™ 120 IR (H^+^) under different conditions of air flow ^1^.

Conditions	Yield, %			Conversion, % ^2^
	TMPMA	TMPDA	TMPTA	
No air flow	25	54	16	60
Venting	26	54	17	61
Bubbling	3	31	65	87

^1^ At 120 °C, 4 h, acid:alcohol 3:1 mol:mol, cat. 10% *w*/*w*_tot_, MEHQ, 0.1% mol/mol_AA_. ^2^ In terms of esterified -OH groups.

**Table 2:** Catalytic tests using Amberlite™ 120 IR (H^+^) under different acid:alcohol ratios ^1^.

Conditions	Acid:Alcohol mol:mol	Yield, %			Conversion, % ^2^
		TMPMA	TMPDA	TMPTA	
No air flow	3:1	25	54	16	60
No air flow	6:1	15	55	31	72
No air flow	9:1	11	26	62	83
Bubbling	3:1	3	31	65	87
Bubbling	6:1	<1	7	92	97
Bubbling	9:1	<1	1	98	99

^1^ At 120 °C, 4 h, cat. 10% *w*/*w*_tot_, MEHQ, 0.1% mol/mol_AA_. ^2^ In terms of esterified -OH groups.

**Table 3:** Catalytic tests using different heterogeneous catalysts ^1^.

Catalyst	Time, *h*	Yield, %			Conversion, % ^2^
		TMPMA	TMPDA	TMPTA	
Dowex™ 50WX8	0.25	54	16	3.1	32
	0.50	46	42	8.1	52
	4.00	<1	29	71	90
Amberlyst^®^ 15	0.25	49	37	4.0	45
	0.50	28	59	10	59
	4.00	<1	<1	>99	>99
Amberlite™ 120 IR (H^+^)	0.25	55	17	2.9	33
	0.50	43	43	9.9	53
	4.00	<1	8	92	98

^1^ At 120 °C, 4 h, acid:alcohol 6:1 mol/mol, cat. 10% *w*/*w*_tot_, MEHQ, 0.1% mol/mol_AA,_ bubbling system. ^2^ In terms of esterified -OH groups.

**Table 4:** Catalytic tests using pre-dried and not pre-dried Amberlite™ 120 IR (H^+^) ^1^.

Conditions	Acid:Alcohol mol:mol	Yield %			Conversion % ^2^
		TMPMA	TMPDA	TMPTA	
Not pre-dried	3:1	3	31	65	87
Not pre-dried	6:1	<1	7	92	97
Not pre-dried	9:1	<1	1	98	99
Pre-dried	3:1	6	34	61	85
Pre-dried	6:1	<1	8	92	98
Pre-dried	9:1	<1	<1	>99	>99

^1^ At 120 °C, 4 h, cat. 10% *w*/*w*_tot_ (based on wet masses), MEHQ, 0.1% mol/mol_AA_, bubbling system. ^2^ In terms of esterified -OH groups.

**Table 5:** Catalytic tests ^1^ using Amberlite™ 120 IR (H^+^) at different loadings ^1^.

Loading *w*/*w*_tot_	Time, *h*	Yield %			Conversion % ^2^
		TMPMA	TMPDA	TMPTA	
	0.25	55	17	2.9	33
10%	0.50	43	43	9.9	53
	4.00	<1	8	92	98
	0.25	36	6.0	<1	16
5.0%	0.50	53	17	3.2	32
	4.00	<1	18	81	93
	0.25	22	1.6	<1	9.0
2.5%	0.50	44	10	2.3	24
	4.00	<1	38	61	86

^1^ At 120 °C, 4 h, acid:alcohol 6:1 mol:mol, MEHQ, 0.1% mol/mol_AA_, bubbling system. ^2^ In terms of esterified -OH groups.

**Table 6:** Catalytic tests using Amberlite™ 120 IR (H^+^) with a mechanical stirrer (400 rpm) under different acid:alcohol ratios. ^1^

Conditions	Acid:Alcohol mol:mol	Yield %			Conversion % ^2^
		**TMPMA**	**TMPDA**	**TMPTA**	
Bubbling	3.9:1	2	72	25	73
Bubbling	4.5:1	9	52	38	76
Bubbling	6:1	5	42	54	83

^1^ At 120 °C, 4 h, cat. 10% *w*/*w*_tot_, MEHQ, 0.1% mol/mol_AA_, bubbling system. ^2^ In terms of esterified -OH groups.

## Data Availability

The raw data supporting the conclusions of this article will be made available by the authors on request.

## Supplementary Materials

The following supporting information can be downloaded at: https://www.mdpi.com/article/10.3390/molecules29040918/s1, Table S1: Protic ion exchange resin characteristics; Figure S1: ^1^H NMR spectra over reaction time; Figure S2: Relevant portion of ^1^H NMR over reaction time; Figure S3: ^1^H NMR spectra of the reaction mixture after 4 h in optimized conditions and acrylic acid distillation (bottom, red), and commercial TMPTA (top, cyan); Figure S4: ^1^H NMR of distilled acrylic acid after reaction time; Figure S5: ^1^H NMR of commercial TMPTA (Frimpeks TR, Turkey) used as reference 100 mg in 600 μL CDCl_3_. Estimated composition: 96% TMPTA, 4% TMPDA; Figure S6: ^13^C NMR of commercial TMPTA (Frimpeks TR, Turkey,) used as reference, 100 mg in 600 μL CDCl_3_. Estimated composition: 96% TMPTA 4% TMPDA; Figure S7. Reaction setup scheme with magnetic stirrer; Figure S8: Left, reaction apparatus overview with a mechanical stirrer; right, details of mechanical stirrer used; Figure S9. Example of ^1^H NMR spectra analysis: integration and signal deconvolution.

## References

[1] Jin X., Meng K., Zhang G., Liu M., Song Y., Song Z., Yang C. (2021). Interfacial catalysts for sustainable chemistry: Advances on atom and energy efficient glycerol conversion to acrylic acid. Green Chem..

[2] Sun D., Yamada Y., Sato S., Ueda W. (2017). Glycerol as a potential renewable raw material for acrylic acid production. Green Chem..

[3] Hermens J.G.H., Jensma A., Feringa B.L. (2022). Highly Efficient Biobased Synthesis of Acrylic Acid. Angew. Chem. Int. Ed..

[4] Matsuura Y., Onda A., Ogo S., Yanagisawa K. (2014). Acrylic acid synthesis from lactic acid over hydroxyapatite catalysts with various cations and anions. Catal. Today.

[5] Bhagyashri P., Pratik M., Eswara P. Acrylic Acid Market Outlook—2021–2030. https://www.alliedmarketresearch.com/acrylic-acid-market.

[6] Mori H., Müller A.H.E. (2003). New polymeric architectures with (meth)acrylic acid segments. Prog. Polym. Sci..

[7] Nollenberger K., Albers J. (2013). Poly(meth)acrylate-based coatings. Int. J. Pharm..

[8] Corsaro C., Neri G., Santoro A., Fazio E. (2022). Acrylate and Methacrylate Polymers’ Applications: Second Life with Inexpensive and Sustainable Recycling Approaches. Materials.

[9] Lin X., Liao B., Zhang J., Li S., Huang J., Pang H. (2019). Synthesis and characterization of high-performance cross-linked polycarboxylate superplasticizers. Constr. Build. Mater..

[10] Petrov P., Bozukov M., Burkhardt M., Muthukrishnan S., Müller A.H.E., Tsvetanov C.B. (2006). Stabilization of polymeric micelles with a mixed poly(ethylene oxide)/poly(2-hydroxyethyl methacrylate) shell by formation of poly(pentaerythritol tetraacrylate) nanonetworks within the micelles. J. Mater. Chem..

[11] Duffy D.J., Das K., Hsu S.L., Penelle J., Rotello V.M., Stidham H.D. (2002). Binding Efficiency and Transport Properties of Molecularly Imprinted Polymer Thin Films. J. Am. Chem. Soc..

[12] Son Y.J., Kim S.J., Kim Y.-J., Jung K.-H. (2020). Selective Vapor Permeation Behavior of Crosslinked PAMPS Membranes. Polymers.

[13] Fertier L., Ibert M., Buffe C., Saint-Loup R., Joly-Duhamel C., Robin J.J., Giani O. (2016). New biosourced UV curable coatings based on isosorbide. Prog. Org. Coat..

[14] Kaczmarek H., Nowicki M., Vuković-Kwiatkowska I., Nowakowska S. (2013). Crosslinked blends of poly(lactic acid) and polyacrylates: AFM, DSC and XRD studies. J. Polym. Res..

[15] Hatanaka L.C., Wang Q., Cheng Z., Mannan M.S. (2017). Effect of trimethylolpropane triacrylate cross-linkages on the thermal stability and char yield of poly (methyl methacrylate) nanocomposites. Fire Saf. J..

[16] Venkatachalam D., Vediappan V., Kaliappa gounder S. (2013). Synthesis and evaluation of trimethylolpropane triacrylate crosslinked superabsorbent polymers for conserving water and fertilizers. J. Appl. Polym. Sci..

[17] Melchiorre M., Esposito R., Russo V., Di Serio M., Elena Cucciolito M., Ruffo F. (2022). Solvent-free direct esterification of acrylic acid with 2-ethylhexyl alcohol using simple Zn(II) catalysts. Inorganica Chim. Acta.

[18] Grzesik M., Skrzypek J., Witczak M., Froment G.F., Waugh K.C. (1999). Kinetics of esterification of acrylic acid with C3 and C4 aliphatic alcohols in the presence of sulfuric acid as a catalyst. Studies in Surface Science and Catalysis.

[19] Jyoti G., Soni R. (2023). Kinetics study of esterification reaction of acrylic acid with n-butanol. Mater. Today Proc..

[20] Jyoti G., Keshav A., Anandkumar J. (2016). Experimental and Kinetic Study of Esterification of Acrylic Acid with Ethanol Using Homogeneous Catalyst. Int. J. Chem. React. Eng..

[21] Jiang W., Jin F.-L., Park S.-J. (2012). Synthesis of ditrimethylolpropane acrylate with low functionality for UV-curable coatings. J. Ind. Eng. Chem..

[22] Sert E., Atalay F.S. (2012). Esterification of Acrylic Acid with Different Alcohols Catalyzed by Zirconia Supported Tungstophosphoric Acid. Ind. Eng. Chem. Res..

[23] Dupont P., Védrine J.C., Paumard E., Hecquet G., Lefebvre F. (1995). Heteropolyacids supported on activated carbon as catalysts for the esterification of acrylic acid by butanol. Appl. Catal. A Gen..

[24] Mekala M., Goli V.R. (2015). Kinetics of esterification of methanol and acetic acid with mineral homogeneous acid catalyst. Chin. J. Chem. Eng..

[25] Lilja J., Murzin D.Y., Salmi T., Aumo J., Mäki-Arvela P., Sundell M. (2002). Esterification of different acids over heterogeneous and homogeneous catalysts and correlation with the Taft equation. J. Mol. Catal. A Chem..

[26] Melchiorre M., Lentini D., Cucciolito M.E., Taddeo F., Hmoudah M., Di Serio M., Ruffo F., Russo V., Esposito R. (2022). Sustainable Ketalization of Glycerol with Ethyl Levulinate Catalyzed by the Iron(III)-Based Metal-Organic Framework MIL-88A. Molecules.

[27] Cucciolito M.E., Di Serio M., Esposito R., Melchiorre M., Ruffo F., Russo V., Tesser R., Turco R., Vitiello R. (2023). Catalysis for Oleochemical Platforms. Eur. J. Inorg. Chem..

[28] Taddeo F., Vitiello R., Tesser R., Melchiorre M., Eränen K., Salmi T., Russo V., Di Serio M. (2022). Nonanoic acid esterification with 2-ethylhexanol: From batch to continuous operation. Chem. Eng. J..

[29] Esposito R., Cucciolito M.E., D’Amora A., Di Guida R., Montagnaro F., Ruffo F. (2017). Highly efficient iron(III) molecular catalysts for solketal production. Fuel Process. Technol..

[30] Komoń T., Niewiadomski P., Oracz P., Jamróz M.E. (2013). Esterification of acrylic acid with 2-ethylhexan-1-ol: Thermodynamic and kinetic study. Appl. Catal. A Gen..

[31] Li R., Schork F.J. (2006). Modeling of the Inhibition Mechanism of Acrylic Acid Polymerization. Ind. Eng. Chem. Res..

[32] Keshipour S., Houshyar M., Fatemi Z. (2019). Synthesis of some Polyol Esters and Diesters Catalyzed with SnO2 and Nano-SnO2. Iran. J. Chem. Chem. Eng..

[33] Khan Z., Javed F., Shamair Z., Hafeez A., Fazal T., Aslam A., Zimmerman W.B., Rehman F. (2021). Current developments in esterification reaction: A review on process and parameters. J. Ind. Eng. Chem..

[34] Leite M.J.L., Marques I.R., Proner M.C., Araújo P.H.H., Ambrosi A., Di Luccio M. (2023). Catalytically active membranes for esterification: A review. Chin. J. Chem. Eng..

[35] Li S., Jiang S., Zhang P., Jiang P., Leng Y. (2022). Protonic ionic liquids as efficient phase-separation catalysts for esterification of trimethylolpropane and acrylic acid. J. Mol. Liq..

[36] Zhang M., Li S., Zhang P., Leng Y. (2023). Sulfonated phenylamine ionic liquids as efficient phase-separation catalysts for the synthesis of trimethylolpropane triacrylate. New J. Chem..

[37] Shashkova V.T., Pevtsova L.A., Zapadinskii B.I., Sokolov V.I., Sister V.G., Ivannikova E.M. (2012). Synthesizing the components of photopolymerizing acryl composites for production of waveguides with high transparency within telecommunication spectral regions. Theor. Found. Chem. Eng..

[38] Nakatake D., Yazaki R., Ohshima T. (2016). Chemoselective Transesterification of Acrylate Derivatives for Functionalized Monomer Synthesis Using a Hard Zinc Alkoxide Generation Strategy. Eur. J. Org. Chem..

