# Supplementary material for: Heterogeneous Brønsted Catalysis in the Solvent-Free and Multigram-Scale Synthesis of Polyalcohol Acrylates: The Case Study of Trimethylolpropane Triacrylate
Source: Molecules. 2024 Feb 19;29(4):918. doi: 10.3390/molecules29040918 (PMC10892049; doi:10.3390/molecules29040918)
Supplement: Supplementary file 1 [file molecules-29-00918-s001.zip › molecules-2843736-supplementary.pdf]

Supplementary Materials

Heterogeneous Brønsted catalysis in the solvent-free and multigram-scale synthesis of polyalcohol acrylates: the case study of trimethylolpropane triacrylate

Massimo Melchiorre[1,2], Maria E. Cucciolito[1,3], Roberto Esposito[1,3], Simone Silvestro[1], Francesco Ruffo[1,3,*]

[1] Dipartimento di Scienze Chimiche, Università di Napoli Federico II, Complesso Universitario di Monte S. Angelo, via Cintia 21, 80126 Napoli (Italy); massimo.melchiorre@unina.it; cuccioli@unina.it; roberto.esposito@unina.it; simonesilvestro01@gmail.com

[2] ISUSCHEM srl, Piazza Carità 32, 80134 Napoli (Italy)

[3] Consorzio Interuniversitario di Reattività Chimica e Catalisi (CIRCC), via Celso Ulpiani 27, 70126 Bari (Italy)

[*] Correspondence: ruffo@unina.it

Table of Contents

Table S1. Protic ion exchange resin characteristicsa. 2
Figure S1. 1H NMR spectra over reaction time. 3
Figure S2. Relevant portion of 1H NMR over reaction time. 3
Figure S3. 1H NMR spectra of the reaction mixture after 4 h in optimized conditions and acrylic acid distillation (bottom, red), and commercial TMPTA (top, cyan). 4
Figure S4. 1H NMR of distilled acrylic acid after reaction time. 4
Figure S5. 1H NMR of commercial TMPTA (Frimpeks TR, Turkey) used as reference 100 mg in 600 ◎L CDCl3. Estimated composition: 96% TMPTA, 4% TMPDA. 5
Figure S6. 13C NMR of commercial TMPTA (Frimpeks TR, Turkey,) used as reference, 100 mg in 600 ◎L CDCl3. Estimated composition: 96% TMPTA 4% TMPDA. 5
Figure S7. Reaction setup scheme with magnetic stirrer. 6
Figure S8. Left, reaction apparatus overview with a mechanical stirrer; right, details of mechanical stirrer used. 7
Figure S9. Example of 1H NMR spectra analysis: integration and signal deconvolution. 8

Table S1. Protic ion exchange resin characteristics [a].

	Amberlite™ 120 IR (H^+)	Amberlyst® 15	DOWEX™ 50WX8	Units
Particle size	28-35	>50	50-100	mesh
	420-590	<300	150-300	μm
Matrix copolymer	Styrene-divinylbenzene (gel)	Styrene-divinylbenzene (macroreticular)	Styrene-divinylbenzene (gel)	–
Crosslinking degree	8	20	8	%
Water retention capacity	53-58	5	50-56	%
Total exchange capacity	1.8	1.7	1.1	meq/mL

[a] Physical form: beads; ionic form: H^+ (functional group: sulfonic acid)

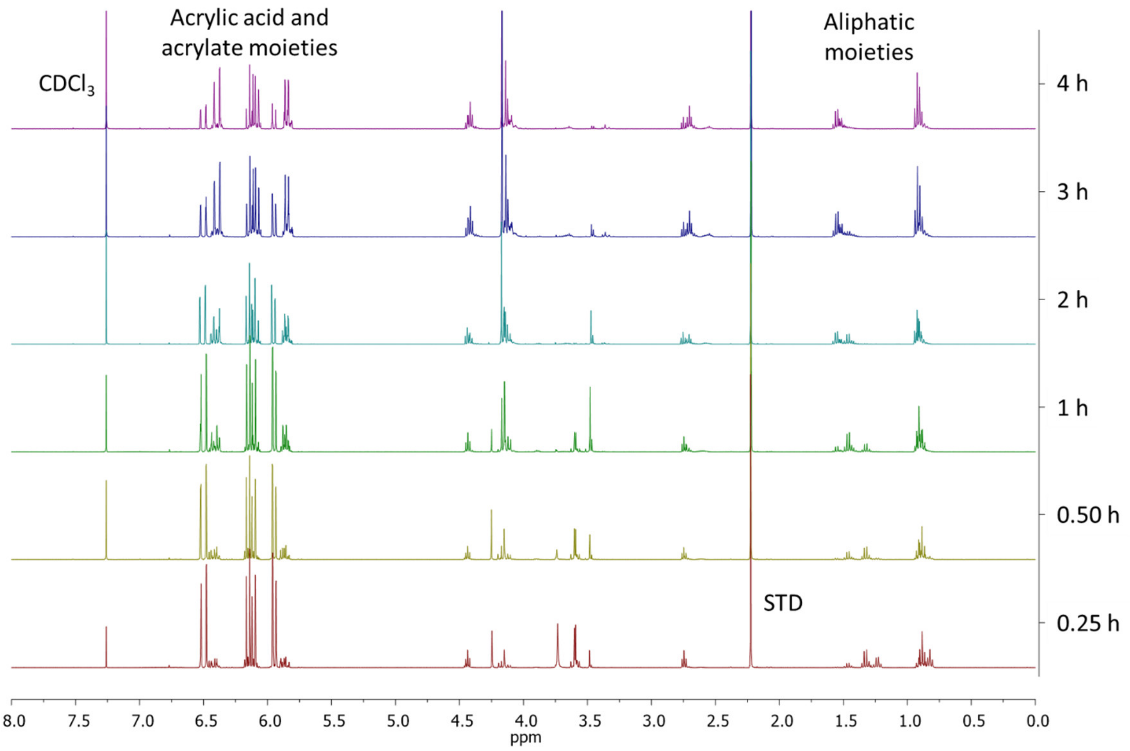

Figure S1. ^{1}H NMR spectra over reaction time.

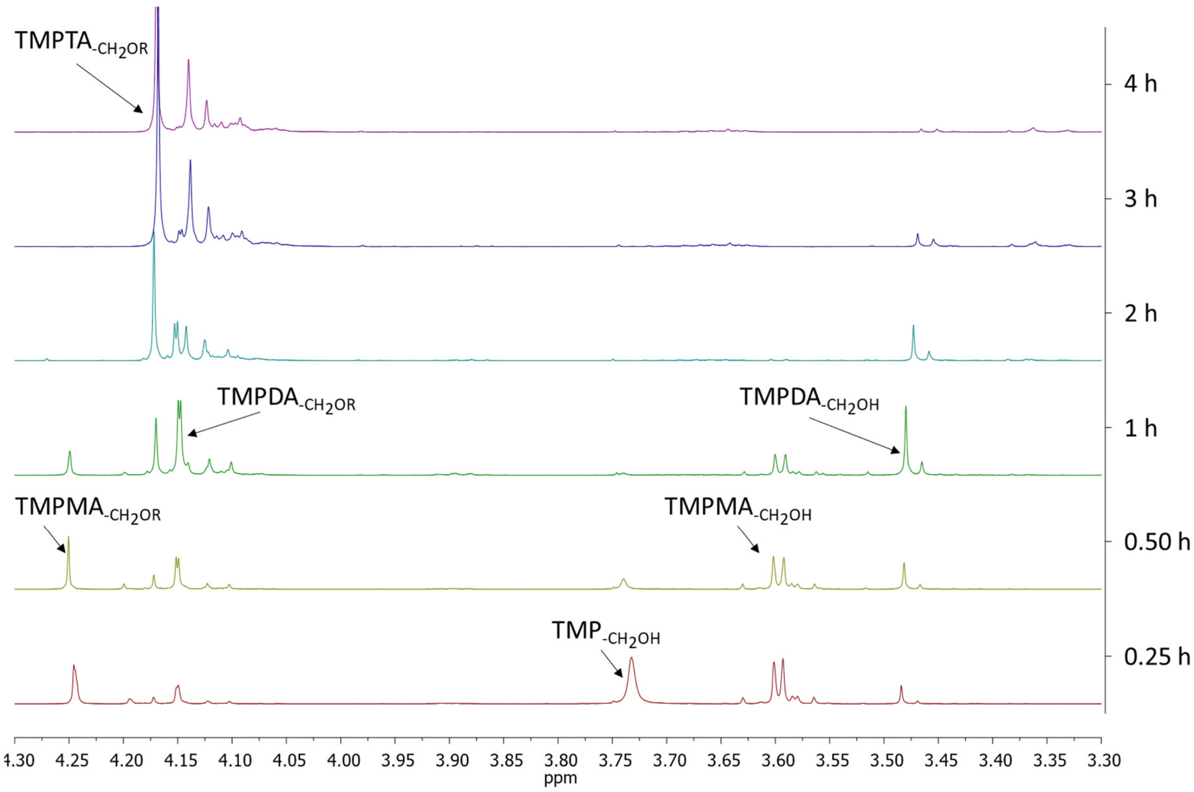

Figure S2. Relevant portion of ^{1}H NMR over reaction time.

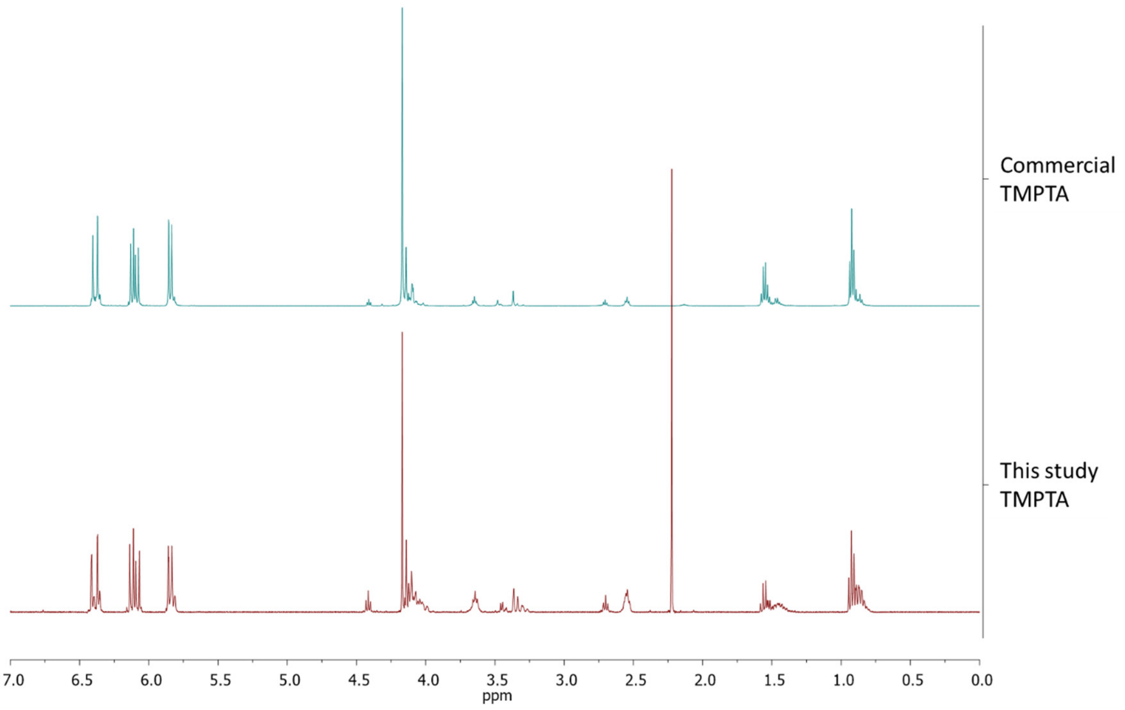

Figure S3. ^{1}H NMR spectra of the reaction mixture after 4 h in optimized conditions and acrylic acid distillation (bottom, red), and commercial TMPTA (top, cyan).

Signals at 4.42, 3.64, 2.70, 2.55 ppm (common in both spectra) are relative to acrylic contaminants: acrylic acid esteric dimers, and β-hydroxyacids (water addition to acrylic acid), which are ubiquitous contaminants in aged acrylic acid.

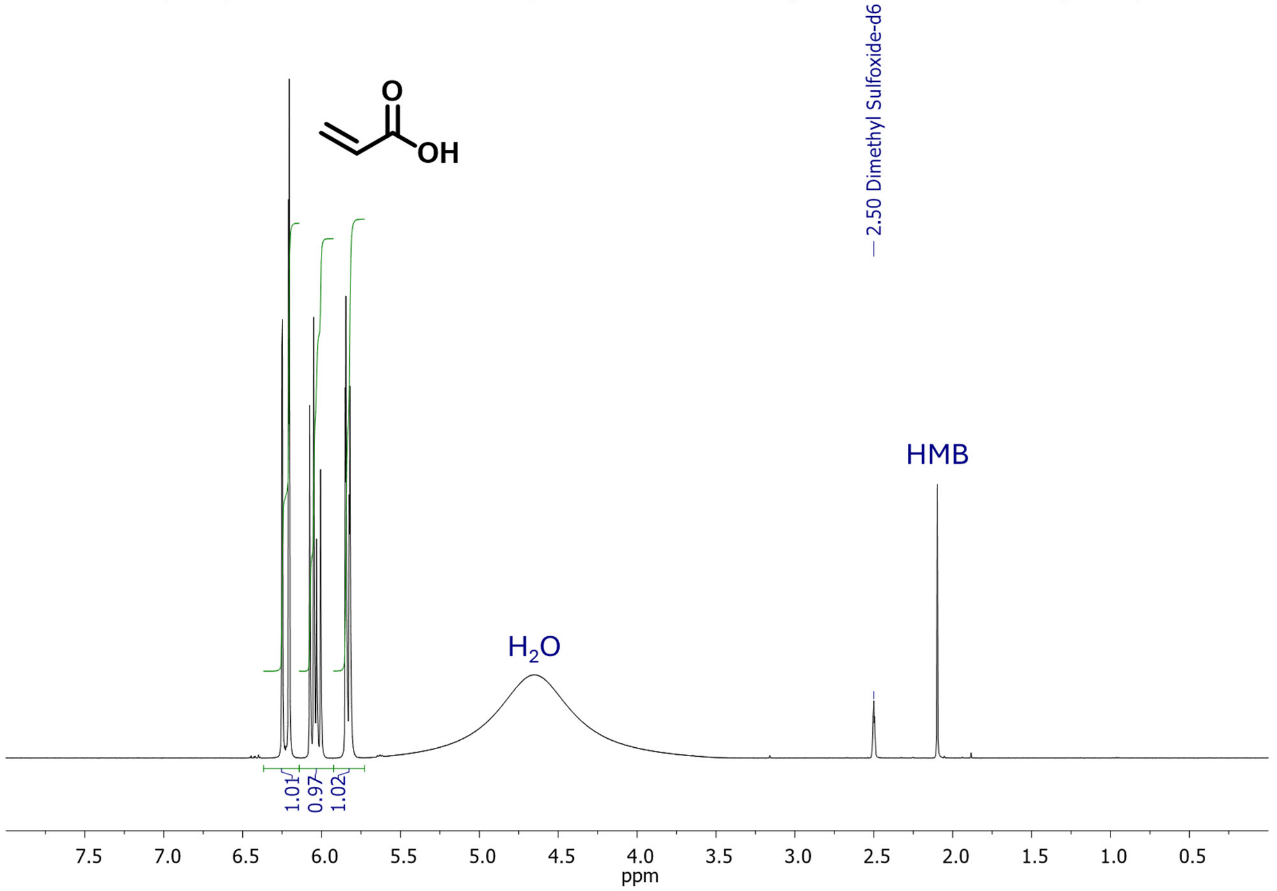

Figure S4. ^{1}H NMR of distilled acrylic acid after reaction time.

^{1}H NMR (400 MHz, DMSO) δ 6.23 (dd, *J* = 17.3, 1.7 Hz, 1H), 6.04 (dd, *J* = 17.3, 10.3 Hz, 1H), 5.83 (dd, *J* = 10.3, 1.7 Hz, 1H).

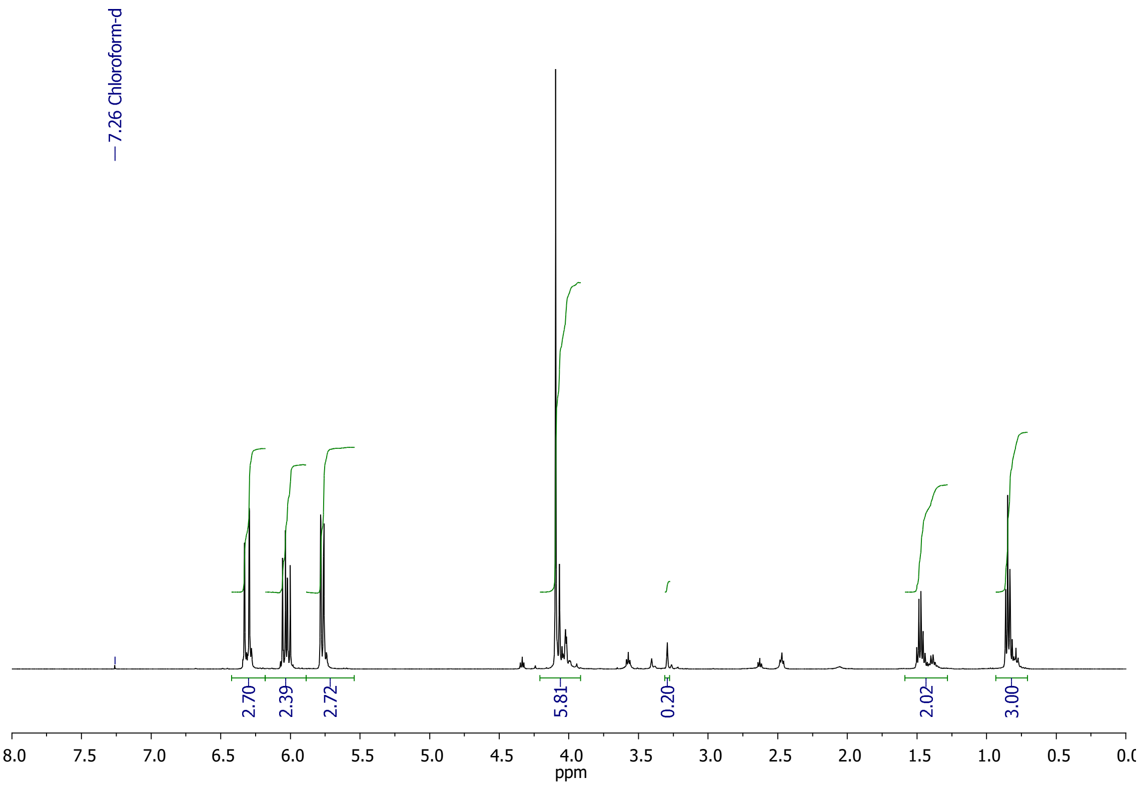

Figure S5. ^{1}H NMR of commercial TMPTA (Frimpeks TR, Turkey) used as reference 100 mg in 600 µL $CDCl_3$. Estimated composition: 96% TMPTA, 4% TMPDA.

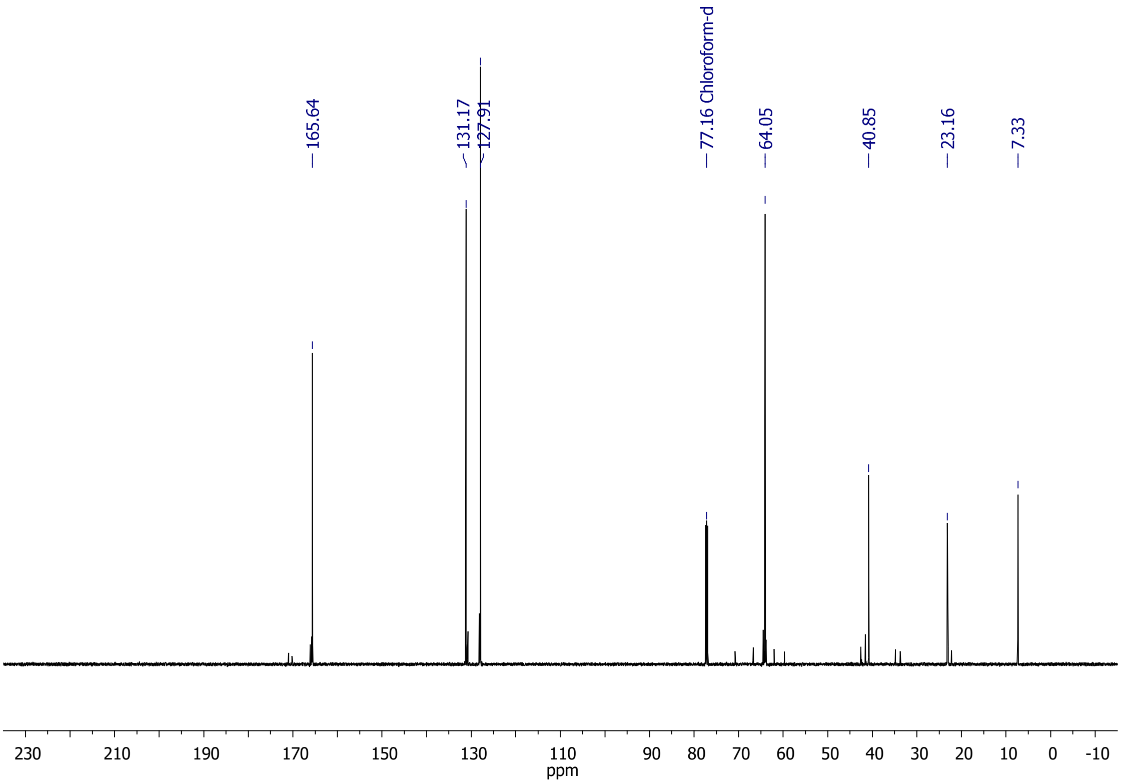

Figure S6. ^{13}C NMR of commercial TMPTA (Frimpeks TR, Turkey,) used as reference, 100 mg in 600 µL $CDCl_3$. Estimated composition: 96% TMPTA 4% TMPDA.

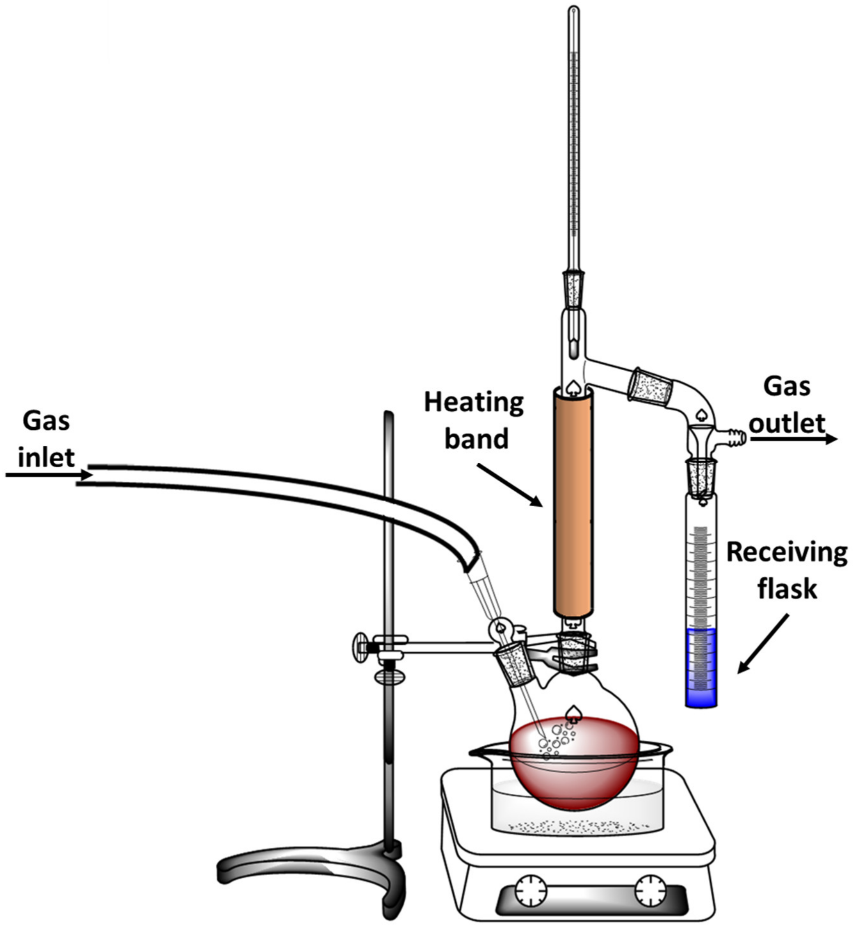

Figure S7. Reaction setup scheme with magnetic stirrer.

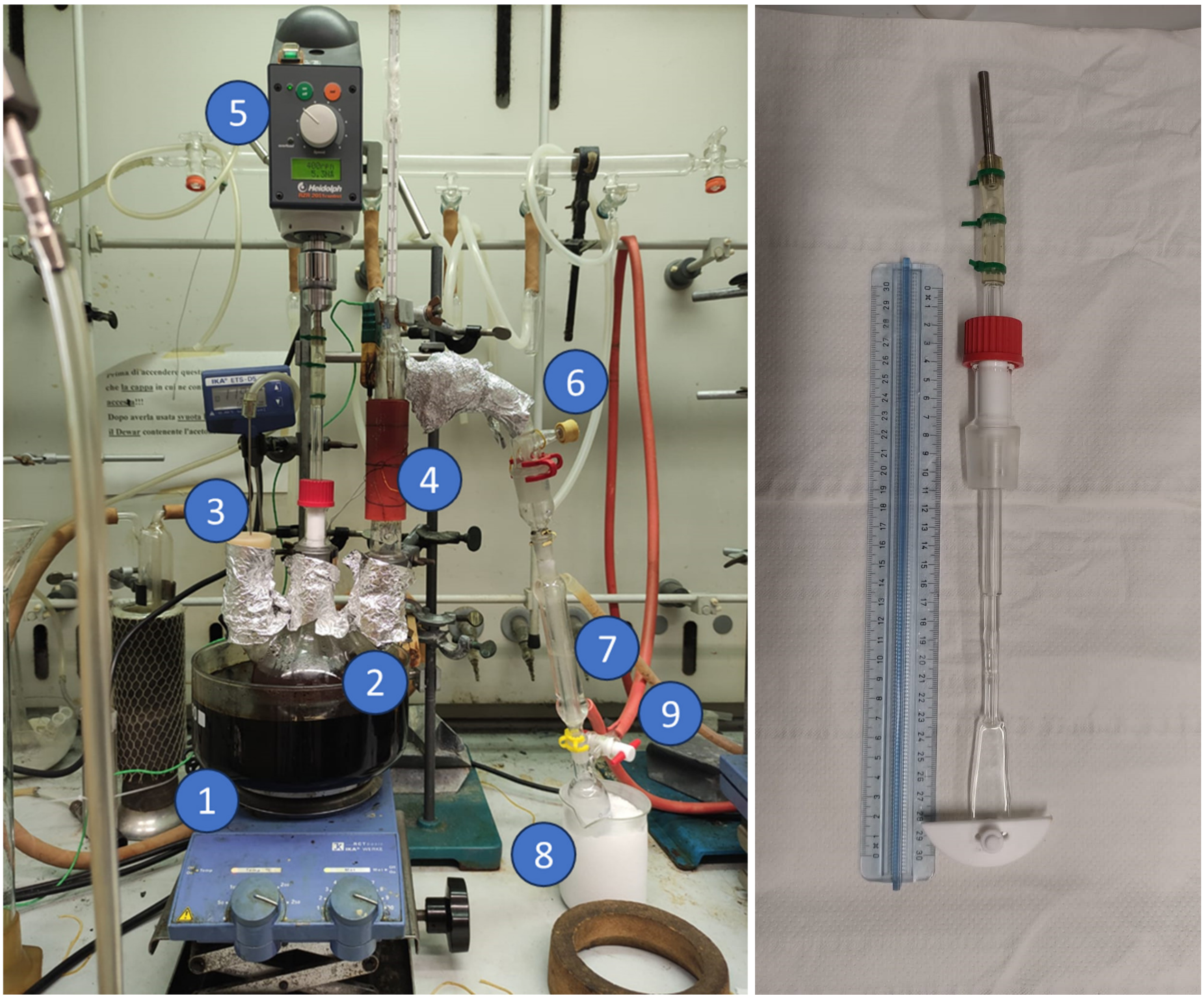

Figure S8. Left, reaction apparatus overview with a mechanical stirrer; right, details of mechanical stirrer used.
1) Heating plate; 2) round-bottom flask; 3) purging needle; 4) heating band; 5) mechanical stirrer; 6) vacuum hose connection (not connected in the picture); 7) water-condenser; 8) receiving flask in dry-ice bath; 9) vapor outlet hose.

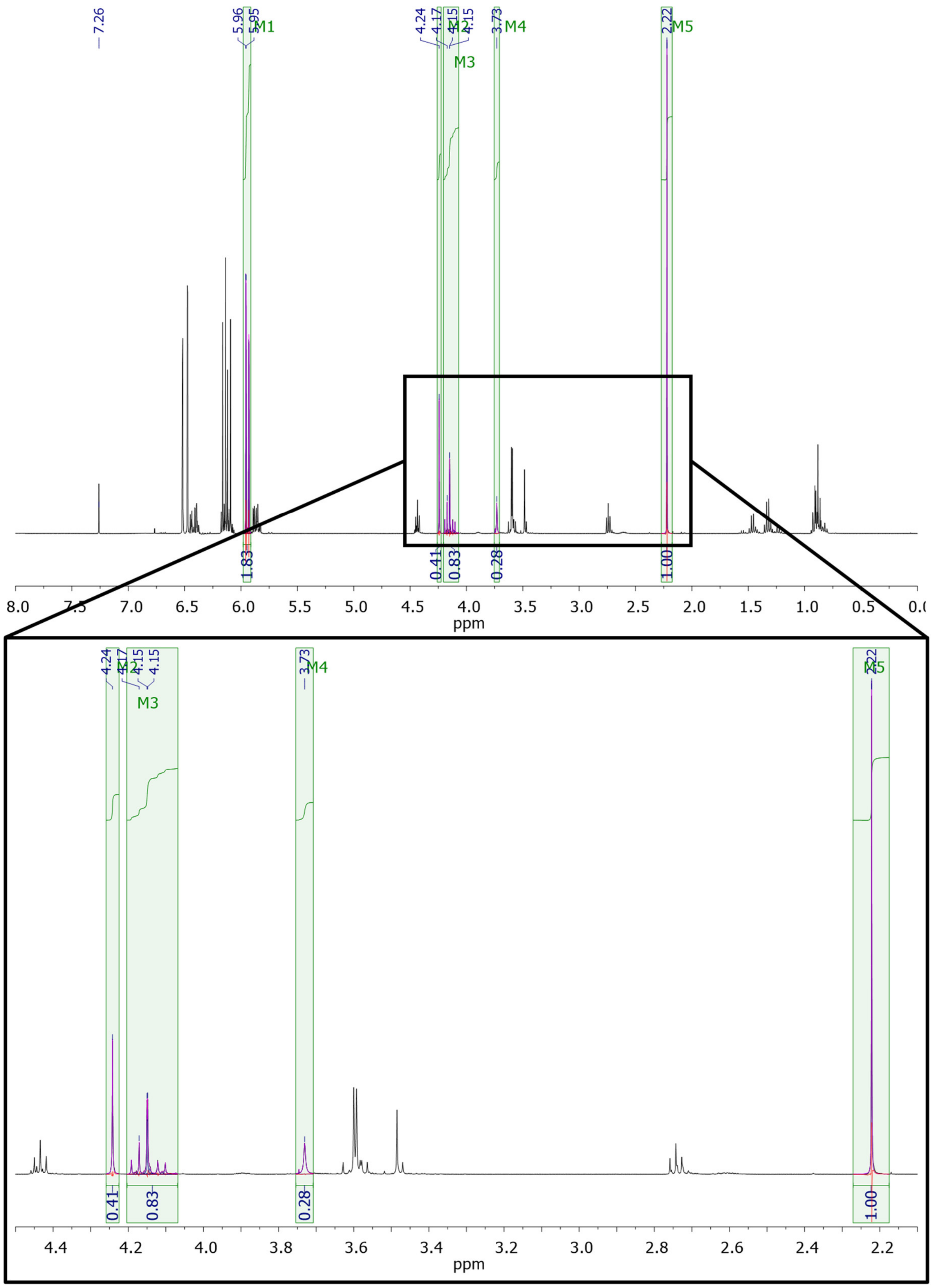

Figure S9. Example of ^{1}H NMR spectra analysis: integration and signal deconvolution.

A sample withdrawn form the reaction mixuture after 30 min is diluted in $CDCl_3$ and analysed by ^{1}H NMR by the addition of a standard solution (hexamethylbenzene 0.1 M in $CDCl_3$). Here is reported the spectrum obtained (reaction conditions: cat IR 120 10%$_{wt}$, molar ratio TMP to AA 1 : 6, stripping condition, 0.5 h, 120 °C).
